# Supplementary material for: Improved and optimized drug repurposing for the SARS-CoV-2 pandemic
Source: PLoS One. 2023 Mar 16;18(3):e0266572. doi: 10.1371/journal.pone.0266572 (PMC10019610; doi:10.1371/journal.pone.0266572)
Supplement: S1 File — (DOCX) [file pone.0266572.s001.docx]

Our implementations of the experiments and the model can be found in

https://doi.org/10.5281/zenodo.7104738
