# Supplementary material for: Improved and optimized drug repurposing for the SARS-CoV-2 pandemic
Source: PLoS One. 2023 Mar 16;18(3):e0266572. doi: 10.1371/journal.pone.0266572 (PMC10019610; doi:10.1371/journal.pone.0266572)
Supplement: S1 Appendix — (PDF) [file pone.0266572.s002.pdf]

Appendix

	Drug Name	KMeans Union	Louvain Max	Mean-Outlier	Drug Clusters	Biclique Union
1	Cyclosporine	✓	✓	✓	✓	✓
2	Sirolimus	✓	✓	✓	✓	✓
3	Ivermectin	✓	✓	✓	✓	✓
4	Methotrexate	✓	✓	✓	✓	✓
5	Dexamethasone	✓	✓	✓	✓	✓
6	Tafenoquine	✓	✓	✓	✓	✓
7	Hydrocortisone	✓	✓	✓	✓	✓
8	Pentoxifylline	✓	✓	✓	✓	✓
9	Ibuprofen	✓	✓	✓	✓	✓
10	Tretinoin	✓	✓	✓	✓	✓
11	Nitrogen	✓	✓	✓	✓	✓
12	Estradiol	✓	✓	✓	✓	✓
13	Colchicine	✓	✓	✓	✓	✓
14	Nitazoxanide	✓	✓	✓	✓	✓
15	Arginine	✓	✓	✓	✓	✓
16	Rosuvastatin	✓	✓	✓	✓	✓
17	Simvastatin	✓	✓	✓	✓	✓
18	Spironolactone	✓	✓	✓	✓	
19	Anakinra	✓	✓	✓		✓
20	Abatacept	✓	✓	✓		✓
21	Interferon beta-1a	✓	✓	✓		✓
22	Hydroxychloroquine	✓		✓	✓	✓
23	Thalidomide		✓	✓	✓	✓
24	Etoposide		✓	✓	✓	✓
25	Crizanlizumab	✓	✓		✓	
26	Nitroglycerin	✓	✓			✓
27	Fluorescein	✓		✓		✓
28	Chlorpromazine		✓	✓	✓	
29	Captopril	✓		✓		
30	Brexanolone	✓				✓
31	Omalizumab	✓				✓
32	Fostamatinib	✓				✓
33	Canakinumab	✓				✓
34	Siltuximab	✓				✓
35	Eculizumab	✓				✓
36	Tacrolimus			✓	✓	
37	Atorvastatin			✓	✓	
38	Fluoxetine		✓			
39	Propranolol		✓			
40	Interferon beta-1b		✓			
41	Cholecalciferol			✓		
42	Metformin			✓		
43	Prednisone				✓	
44	Isotretinoin				✓	
45	Prednisolone				✓	
46	Methylprednisolone				✓	
47	Tamoxifen				✓	

Table 3. Drugs that are already in clinical trials which our model ranks in the top 100.

	Drug Name	KMeans Union	Louvain Max	Mean-Outlier	Drug Clusters	Biclique Union
1	Cyclosporine	3	6	2	2	3
2	Sirolimus	16	20	10	4	13
3	Ivermectin	13	14	6	30	7
4	Methotrexate	19	17	7	29	10
5	Dexamethasone	10	26	12	31	19
6	Tafenoquine	21	7	9	74	21
7	Hydrocortisone	25	41	20	38	27
8	Pentoxifylline	46	50	45	19	22
9	Ibuprofen	73	31	49	17	34
10	Tretinoin	48	53	32	39	39
11	Nitrogen	11	5	74	80	51
12	Estradiol	100	68	35	12	75
13	Colchicine	99	64	40	45	50
14	Nitazoxanide	59	82	22	75	74
15	Arginine	49	21	83	79	81
16	Rosuvastatin	67	88	46	88	69
17	Simvastatin	94	93	56	89	89
18	Spironolactone	89	92	27	10	
19	Anakinra	27	15	50		26
20	Abatacept	33	29	42		32
21	Interferon beta-1a	61	13	87		60
22	Hydroxychloroquine	86		63	52	83
23	Thalidomide		74	30	41	77
24	Etoposide		83	59	51	98
25	Crizanlizumab	60	55		83	
26	Nitroglycerin	84	100			70
27	Fluorescein	64		82		86
28	Chlorpromazine		37	85	94	
29	Captopril	79		95		
30	Brexanolone	2				2
31	Omalizumab	58				57
32	Fostamatinib	66				65
33	Canakinumab	68				68
34	Siltuximab	77				76
35	Eculizumab	80				79
36	Tacrolimus			80	54	
37	Atorvastatin			71	91	
38	Fluoxetine		57			
39	Propranolol		62			
40	Interferon beta-1b		87			
41	Cholecalciferol			89		
42	Metformin			93		
43	Prednisone				63	
44	Isotretinoin				67	
45	Prednisolone				69	
46	Methylprednisolone				70	
47	Tamoxifen				97	

Table 4. Drugs that are already in clinical trials which our model ranks in the top 100, with the rankings given by each strategy.

	Drug Name	KMeans Union	Louvain Max	Mean-Outlier	Drug Clusters	Biclique Union
1	Cidofovir	1	3	1	1	4
2	Valaciclovir	14	8	5	3	20
3	Hydralazine	6	22	13	5	8
4	Tazarotene	8	18	3	28	16
5	Hydroquinone	4	2	4	72	1
6	Bromocriptine	17	11	18	6	42
7	Acyclovir	28	36	17	35	24
8	Finasteride	35	49	33	11	17
9	Betamethasone	34	35	15	32	30
10	Propylthiouracil	23	52	24	7	48
11	Vincristine	45	30	14	34	36
12	Clonidine	40	16	60	15	31
13	Sunitinib	22	33	16	33	58
14	Somatotropin	20	4	51	77	11
15	Nitisinone	29	9	8	73	67
16	Thiamine	65	28	26	9	59
17	Tadalafil	38	48	54	20	28
18	Acetylsalicylic acid	57	67	43	18	14
19	Vinblastine	56	47	21	36	44
20	Pyrimethamine	37	45	19	37	66
21	Pentamidine	31	59	31	43	41
22	Duloxetine	76	27	55	24	25
23	Etacrynic acid	70	75	23	8	64
24	Sulindac	32	78	67	23	54
25	Paclitaxel	69	60	37	44	72
26	Proguanil	54	79	41	47	63
27	Everolimus	75	63	47	48	53
28	Tamsulosin	90	51	61	16	78
29	Cyclophosphamide	91	71	38	46	56
30	Rifapentine	42	69	68	92	47
31	Terbinafine	83	84	53	50	61
32	Fluvastatin	78	96	91	95	95

Table 5. Drugs that are **not** known to us to be already in clinical trials which our model ranks in the top 100. The entry of a column is the rank with respect to that aggregation strategy.
