# Supplementary material for: Improved and optimized drug repurposing for the SARS-CoV-2 pandemic
Source: PLoS One. 2023 Mar 16;18(3):e0266572. doi: 10.1371/journal.pone.0266572 (PMC10019610; doi:10.1371/journal.pone.0266572)
Supplement: Attachment — Submitted filename: PlosRebuttal.pdf [file pone.0266572.s003.pdf]

Dear PLOS ONE editor(s),

Thank you for your interest in publishing our paper and for the detailed and helpful review. We have tried our best to incorporate all your suggestions. Please find below our response to the suggestions.

<<<<<<<<<<<<<

When submitting your revision, we need you to address these additional requirements.

1. Please ensure that your manuscript meets PLOS ONE's style requirements, including those for file naming. The PLOS ONE style templates can be found at

https://journals.plos.org/plosone/s/file?id=wjVg/ and

https://journals.plos.org/plosone/s/file?id=ba62/

>>>>>>>>>>>>>>>>>>>>>>

We have followed the template and the style requirements.

<<<<<<<<<<<<<<<<<<<<<<

2. Please amend the manuscript submission data (via Edit Submission) to include author Andrew Wood3.

>>>>>>>>>>>>>>>>>>>>>>>>>>>>>

We have added this.

<<<<<<<<<<<<<<<<<<<<<<<<<<<<<<<<<

3. Thank you for submitting the above manuscript to PLOS ONE. During our internal evaluation of the manuscript, we found significant text overlap between your submission and the following previously published works, some of which you are an author.

- https://link.springer.com/

We would like to make you aware that copying extracts from previous publications, especially outside the methods section, word-for-word is unacceptable. In addition, the reproduction of text from published reports has implications for the copyright that may apply to the publications.

Please revise the manuscript to rephrase the duplicated text, cite your sources, and provide details as to how the current manuscript advances on previous work. Please note that further consideration is dependent on the submission of a manuscript that addresses these concerns about the overlap in text with published work.

We will carefully review your manuscript upon resubmission, so please ensure that your revision is thorough.

>>>>>>>>>>>>>>>>>>>>>>>>>>>

The above paper is a conference (Complex Networks) submission and the current submitted paper is the Journal version of this paper. In fact, we were invited to submit for the special issue of Complex Networks. So, we think it is natural that there is some overlap with the conference version. We have included 30% additional material as part of the journal submission.

<<<<<<<<<<<<<<<<<<<<<<<

Additional Editor Comments:

I read the article myself and I read also the Reviewer 1 comments.

As an editor I suggest the following:

1. If the Authors want to propose a novel approach of drug repurposing.

>>>>>>>>>>>>>>>>>>>>>>>>>>>>>>>>>>>>>>>

Our approach has both novel and re-implementation components. The novel components are the aggregation methods which lead to considerable improvement in the post-processing phase, increasing by more than 50% the number of predicted drugs

in the top-100 that were or are in clinical trials. For example, we considered the following list of new aggregation strategies for drug repurposing, all of which are described in the paper: Global Score Maximum, Global Score Mean, Mean with outliers, K-Means cluster Score Maximum, Union over k-means cluster Rankings, Louvain cluster Score Maximum, Drug clusters mean with KMeans, Biclique Max, Union over Biclique rankings. We also list the new drugs that we discovered, where the first drug in our list has recently been added for clinical trials [2]

In addition to novelty, our approach also includes re-implementation. We re-implement the model described by Doshi et al. [1] and improve it by allowing flexible neighborhood capture sizes. Moreover, we improve the overall code quality by following PyTorch idioms and replacing manual calculations with implementations from the scikit-learn library. We also improve the training speed, inference time, readability and by reducing pre-processing time from 30 minutes to 2 minutes by leveraging matrix operations. In addition, we implement our novel aggregation strategies, and also implement some of them using the new NVRAM hardware and show that NVRAM can accelerate the post-processing for drug repurposing.

<<<<<<<<<<<<<<<<<<<<<<<<<<<<<<<<<<<<<<

I agree with Reviewer 1 that, in this case authors should compare the approach with respect state of the art, while they compare only with a previous approach of the same group.For instance, if applicable, the authors can consider to compare with other methods in literature that are of relevance and reported here:

Network medicine framework for identifying drug-repurposing opportunities for COVID-19
Deisy Morselli Gysi et al. PNAS 2021

Pioneering topological methods for network-based drug–target prediction by exploiting a brain-network self-organization theory
C Durán, S Daminelli, JM Thomas, VJ Haupt, M Schroeder, …
Briefings in bioinformatics 19 (6), 1183-1202

I want to stress that to accept your study, I do not need that your proposed method performs better than the ones you compare .I only require a fair comparison that shows and discusses the level of performance in respect with other methods that are considered state of art in literature.This is important to evaluate your proposed method in the context of the current scientific literature.

>>>

Thank you for your suggestions. We compared our work with the first paper that you suggested. We have included the results in the paper. For the second one, we think it is not applicable, as they do not perform experiments for COVID-19. Also, we could not find a code repository for this work, which makes it out of the scope to do this for this revision.

<<<<<<<<<<<<<<<<<<<<<<<<<<<<<<<<<<<<<<<<<<<<<

2. If the Authors want to present an efficient and novel implementation of a previous approach.>>>>>>>>>>>>>>>>>>>>>>>>>>>

We re-implement the previous approach by Doshi et al.[1] using more parallelism and faster training. Our implementation heavily makes use of the python idioms to achieve parallelism and reduce the training time.

<<<<<<<<<<<<<<<<<<<<<

I disagree with Reviewer 1 that in this case probably PLOS one is not the best option (since authors should present advancements in computer science and high performance calculus).I believe that a new efficient implementation of a previous approach is of interest for our journal if the Authors can provide/discuss quantitative and relevant evidence at support of the new implementation in comparison to the previous, but also compare with other methods not considered in previous study of the same group, such as the one I mentioned at the point above.

>>>>>>>>>>>>>>>>>>>>>>>>>>>>>>>>>

We have made comparisons with one of the suggested works, the other one was out of scope as explained above.

<<<<<<<<<<<<<<<<<<<<<<<<<<<<<<<<<<<<<<<

3. I agree with Reviewer 1 that the authors should clarify the context in the introduction and then to modify results and discussion consequently, since they did a hard work, and we are interested to publish their article.

Reviewer #1: Authors present an interesting approach for drug repurposing. The approach is based on graph neural network, python programming language and high performance platforms. The approach is technically sound and it is nicely written. References are also adequate. Despite this, in my opinion paper has a main weakness due to lack of the clarity of the message. Do the authors present a novel approach of drug repurposing? . In this case authors should compare the approach with respect state of the art, while they compare ongly with a previous approach of the same group. So in this case paper should be rejected. Alternatively, the authors want to present an efficient and novel implementation of a previous approach. In this case probably PLOS one is not the best option, since authors should present advancements in computer science and high performace calculus. I know that this a common problem in all the multidisciplinary work, so i suggest to the authors to clarify the context in the introduction and then to modify results and discussion consequently, since they did a hard work.

>>>>>>>>>>>>>>>>>>>>>

As mentioned before, our approach has both novel and re-implementation components. The novelty is mainly in the post-processing phase, more specifically, we introduce new aggregation strategies to get predictions that are better by about 50%, in comparison to both [1] and the paper suggested by the Editor[3]. We also provide a list of drugs that have been predicted with our strategies, with the hope that they can be useful for further biological experimentation. The re-implementation includes re-implementing the model described by Doshi et al. [1], improving the training speed, inference time, readability and by reducing pre-processing time from 30 minutes to 2 minutes by leveraging matrix operations, implementing our novel aggregation strategies, and also accelerating some of them by implementing them using the new NVRAM hardware.

Thanking You,

Davis Issac

[1] Doshi S, Chepuri SP. Dr-COVID: Graph Neural Networks for SARS-CoV-2 Drug Repurposing. CoRR. 2020

[2] Rahman, Mohammad Rejaur, et al. "Identification of potential antivirals against SARS-CoV-2 using virtual screening method." Informatics in medicine unlocked 23 (2021): 100531.

[3] Network medicine framework for identifying drug-repurposing opportunities for COVID-19
Deisy Morselli Gysi et al. PNAS 2021
